# Long-Term Safety of Systemic Ozone Therapy in a Patient With Mitochondrial Encephalomyopathy, Lactic Acidosis, and Stroke-Like Episodes (MELAS)

**DOI:** 10.7759/cureus.48261

**Published:** 2023-11-04

**Authors:** Daniele Romanello, Sara Rotunno, Mauro Martinelli

**Affiliations:** 1 Internal Medicine, Ospedale San Pietro Fatebenefratelli, Roma, ITA; 2 Biomedical Sciences, Ozone Therapy Unit, Ospedale San Pietro Fatebenefratelli, Roma, ITA

**Keywords:** mitochondrial disease, oxidative stress, safety study, melas syndrome, ozone therapy

## Abstract

A patient with mitochondrial encephalomyopathy, lactic acidosis, and stroke-like episodes (MELAS) syndrome, a rare mitochondrial disease characterized by myopathy, epilepsy, encephalopathy, acidosis, and recurrent cerebral ischemic episodes, underwent systemic hematogenous ozone therapy for 17 years. Despite advancements in the study of mitochondrial diseases, there are currently no available treatments for MELAS. The patient in this case has received over 280 sessions of systemic hematic ozone therapy since 2003 (from the age of 10 years) till the time of publication, without reporting any adverse effects, achieving a normal level of development considering the comorbidities. Possible mechanisms of action of systemic hematogenous ozone therapy include improved efficiency of the mitochondrial oxidative chain through the induction of antioxidant enzymes (catalase, superoxide dismutases {SOD}, peroxidase). More studies are needed to evaluate the actual safety of long-term systemic hematogenous ozone therapy in patients with mitochondrial diseases.

## Introduction

Mitochondrial encephalomyopathy, lactic acidosis, and stroke-like episodes (MELAS) is a syndrome characterized by progressive encephalopathy, myopathy, stroke-like episodes, and lactic acidosis, it belongs to the group of mitochondrial diseases, sharing the characteristics of encephalopathy and myopathy [1].

The first suggestive case of MELAS was described in the literature in 1975, where the patient presented mitochondrial myopathy associated with cerebral modifications, such as mental retardation, epilepsy, myoclonus, ophthalmoplegia, retinitis pigmentosa, blindness, basal ganglia calcifications, and sudden hemiplegia suggestive of stroke [2].

Diagnostic criteria for MELAS should include the occurrence of the following events: (1) signs of encephalopathy often associated with dementia and epilepsy, (2) stroke-like episodes in early life, and (3) biochemical evidence of mitochondrial dysfunction, such as acidosis and the presence of ragged-red fibers (RRF) on muscle biopsy [1,2].

MELAS is often associated with the A<G mutation in position 3243 of mitochondrial DNA (mtDNA). The clinical manifestations of this mutation are likely underestimated, and the frequency of the mutation appears to be more prevalent than originally thought [3]. The mutation is present only in a percentage of the mitochondrial DNA in the cell, showing a degree of heteroplasmy that correlates with the age of onset and severity of symptoms [2,3].

In a follow-up study of 33 patients evaluated annually over a period of three years, a low number of new neurological events, progression of sensorineural hearing impairment (SNHI), increased left ventricular thickness, reduced average alpha frequency in occipital and parietal regions, and significant progression of the severity of disease index (modified ranking score) were observed, indicating disease progression [4].

There are no studies in the literature regarding the possibility of treating "MELAS" with ozone therapy. Ozone therapy is a non-invasive, non-pharmacological, and no-side effect procedure. This study aimed to provide a long-term safety report of systemic ozone therapy.

## Case presentation

A 10-year-old patient was admitted in 2003 following the onset of partial epileptic seizures associated with left hemiparesis. Upon admission to the Neurology Department of Bambino Gesù Pediatric Hospital, the child presented with hemiparesis associated with left-sided sensory hemisyndrome. Brain MRI revealed recent ischemic vascular lesions in the right temporoparietal region and a small-sized lesion in the left temporal region. Electroencephalographic examination showed repeated slow-wave abnormalities in the centroparietal regions of the right hemisphere.

After seven days, a repeat radiological examination showed a modest reduction in the area of altered signal intensity of the lesion in the right temporoparietal region, with cortico-subcortical distribution and blurred margins, characterized by hyperintensity in T2 and hypointensity in T1, along with a reduction in the concurrent flattening of adjacent subarachnoid sulci. The examination also revealed a marked increase in the area of altered signal intensity in the left cortico-subcortical temporoparietal region, previously reported in the temporal region.

Laboratory tests showed elevated levels of lactic acid in both cerebrospinal fluid (45 mg/dL, reference range: 10-22 mg/dL) and blood (29.4 mg/dL, reference range: 5-16 mg/dL). Genetic testing performed on mitochondrial DNA confirmed the presence of the MELAS mutation (3243 A<G) in 50% of the mtDNA molecules.

Other laboratory findings, including levels of urinary organic acids, homocysteine, cryoglobulins, rheumatoid factor, complement levels, autoantibodies, and circulating immune complexes, were within normal ranges. No abnormalities were found in the epiaortic vessel Doppler ultrasound, Holter ECG, echocardiogram, audiometric examination, or ophthalmological assessment. Thrombophilia screening was normal. The patient also underwent muscle and skin biopsies, which yielded positive results (documentation not available for review).

The child was discharged with a diagnosis of MELAS and prescribed acetylsalicylic acid 100 mg, coenzyme Q10 400 mg, and carbamazepine 400 mg. Follow-up was scheduled at the Day Hospital of Bambino Gesù Hospital, and further genetic testing was recommended for the maternal lineage and younger brother, who was also found to carry the mutation with a higher percentage of affected molecules.

Over the following eight months, despite regular adherence to the prescribed therapy, the patient experienced six additional hospitalizations due to recurrent exacerbations of the disease, all characterized by epileptic seizures exacerbated by ischemic episodes.

Since 2003, after the last hospitalization, the patient started receiving systemic hematogenous ozone therapy. Prior to each session, glucose-6-phosphate dehydrogenase (G6PDH) levels were measured. The therapy followed the following schedule: 200 ml of O2/O3 mixture at the concentration of 50 mcg/mL in 200 mL of blood, twice a week for four months, then once a week for eight months, once every 14 days for six months, once every 21 days for six months, and once every 28 days thereafter. The patient continued with the scheduled follow-up visits at the Neurology Day Hospital of Bambino Gesù Hospital in Rome, while only taking antiepileptic therapy (carbamazepine 400 mg/day) and discontinuing coenzyme Q10 after the initial six months and acetylsalicylic acid after six months.

In 2011, the patient came under our observation to continue with systemic hematogenous ozone therapy at a frequency of every 28 days, using the same dosages of the oxygen-ozone mixture and blood volume (200 ml at 50 mcg/mL, 200 mL). Throughout the patient's clinical history, they achieved a normal level of development, considering the neurological outcomes resulting from the reported cerebral ischemic events, reaching a height of 160 cm and a weight of 55 kg.

No new ischemic events or epileptic seizures occurred during the observation period. During the period of undergoing systemic hematogenous ozone therapy, the patient did not experience any adverse events.

## Discussion

The purpose of this study is not only to evaluate the effectiveness of ozone therapy in treating patients with MELAS but also to provide a long-term safety report of systemic hematic ozone therapy. In 17 years of therapy, nine of which were under our observation, no adverse effects were reported in a patient with MELAS. Furthermore, the disease progression was not worsened by this procedure. In fact, no ischemic or epileptic events occurred during the observation period when the patient underwent systemic hematic ozone therapy. We also propose this event as a long-term safety report for systemic hematic ozone therapy since, according to the guidelines of the Italian Federation of Ozone Therapy (Nuova FIO) a positive history of epilepsy is included in the recommendations (paragraph 6c) as a precautionary measure, as some experiences have shown the possible triggering of seizures during administration.

Currently, there is no definitive therapy available for MELAS, and any therapeutic intervention aims to provide support [5]. The use of arginine therapy is recommended to manage stroke-like episodes by administering an intravenous bolus (500 mg/kg for children or 10 g/m^2^ of body surface area for adults) within the first three hours of symptom onset, followed by an infusion of the same dose for 24 hours for the next three to five days. After the first stroke-like episode, arginine should be administered as prophylaxis [5,6].

In some individuals, the effectiveness of integrative therapy with coenzyme Q10, L-carnitine, and creatine has been demonstrated [5,7]. Systemic ozone therapy induces a biological response by increasing blood flow and oxygenation of ischemic tissue as a result of the concomitant effect of nitric oxide (NO) and carbon monoxide (CO), intraerythrocytic increase of 2,3-DPG levels, increased levels of reduced glutathione, increased oxidative metabolism induced by enhanced oxygenation, increased cellular antioxidant enzymes, induction of HO-1 and HSP-70, moderate activation of the immune system, and increased release of growth factors [8].

At the mitochondrial level, the 3243 A>G mutation, which is frequently found in patients with MELAS and in the case under analysis, leads to premature termination of transcription, preventing the expression of normal rRNA and causing an alteration in the respiratory chain through a single point mutation in the tRNA-Leu (UUR) gene. This mutation compromises the synthesis of mitochondrial proteins involved in oxidative phosphorylation and ATP production [9,10]. One of the main effects of ozone is the acceleration of glycolysis through the continuous oxidation of NADH molecules, which is crucial for the progression of this process. Additionally, during ozone therapy, there is an increase in transmembrane oxygen flux and induction of enzymes with antioxidant functions, such as superoxide dismutase, peroxidase, or catalase, making oxygen utilization in the mitochondrial respiratory chain more efficient [11].

## Conclusions

This study aimed to document the treatment with systemic ozone therapy over 280 sessions in a patient with MELAS, which has shown no side effects over a period of 17 years. No abnormalities were found in routine examinations, the latest brain MRI is comparable to previous controls, and the patient did not experience epileptic seizures during the observation period. Supporting our findings is the absence of new ischemic events in the disease progression, with the patient only experiencing chronic effects from previous events, including hearing loss, inconsistent language production, intellectual disability, lateralized motor deficits, and insulin-dependent diabetes mellitus. Similarly, the patient's brother also underwent systemic ozone therapy, although it was performed intermittently and discontinued after five years, but with no adverse effects. The patient passed away in 2008. Further studies are needed to investigate the potential efficacy and mechanism of action of this therapy in mitochondrial diseases.
